# The Israeli anesthesiology workforce crisis: a reassessment survey

**DOI:** 10.1186/s13584-024-00620-0

**Published:** 2024-09-17

**Authors:** Ariel Wimpfheimer, Yehuda Ginosar, Shai Fein, Esty Goldberger, Charles Weissman, the Israel Anesthesiology Workforce Study Group, Haled Abd-Al-Halim, Hakeem Abu-Rais, Chaim Berkenstadt, Ilya Chernoy, Maruan Armaly, Yaakov Duvdivani, Leonid Eidelman, Shai Fine, Brian Fredman, Yulia Gadulov, Zeev Goldik, Yaakov Gozal, Zoya Haituv, Alex Izakson, Yaakov Katz, Idit Matot, Noam Mubada, Reuven Pizov, Aeyal Raz, Gefen Revaz, Igor Reznikof, Nogzar Rigzny, Michael Rudin, Vladimir Rukinglass, Albert Sabatnitzki, Eran Segal, Eric Siton, Mustafa Somri, Riad Tome, Jacob Turban, Nathan Weksler, Dafna Wilner, Yossi Witchelevsky, Alex Zlotnik

**Affiliations:** 1 Faculty of Medicine of the Hebrew University of Jerusalem, Jerusalem, Israel; 2 Hospital Administration, Hadassah-Hebrew University Medical Center, Jerusalem, Israel; 3 Department of Anesthesiology and Operating Rooms, Rabin Medical Center, Bellinson Hospital, Petach Tikva, Jerusalem, Israel; 4 Department of Anesthesiology, Critical Care Medicine and Pain Management, Hadassah-Hebrew University Medical Center, Jerusalem, Israel; 5 Braun School of Public Health, Hebrew University of Jerusalem, Jerusalem, Israel; 6 Department of Anesthesiology, Critical Care and Pain Management, Hadassah-Hebrew University Medical Center, Kiryat Hadassah, Jerusalem, POB 12000, 91120 Israel

**Keywords:** Anesthesiology, Israel, Physician workforce, Residency, Workforce shortage

## Abstract

**Background:**

Anesthesiologists provide crucial anesthesiology services in the operating room and non-operating room locations. Combined with an aging and growing Israeli population, there is an increasing demand for anesthesiology services. A previous study performed in 2005 showed that most anesthesiologists are immigrant physicians with few Israeli medical school graduates. Since then, physician immigration decreased, many have retired and demand for anesthesia services has increased while insufficient numbers of new anesthesiologists were trained, leading to a shortage, limiting surgeries and other procedures in many hospitals. The present study examined the composition of the Israeli anesthesiology workforce in 2021and compared it to the 2005 workforce.

**Methods:**

A cross-sectional survey of demographic and professional information about each Israeli hospital anesthesiologists was solicited from 34 anesthesiology department chairs responsible for 36 Israeli acute care hospitals.

**Results:**

There are 1313 anesthesiologists in the 36 hospitals, resulting in a ratio of 14.2 anesthesiologists per 100,000 population. 22.6% of anesthesiologists will reach retirement age over the next ten years. The proportion of female anesthesiologists was 28.7%. While Israeli medical school graduates increased to 18.1% from 12.2% in 2005, non-Israeli citizens and non-permanent residents comprised 8.5% of the workforce.

**Conclusions:**

Despite growth in the ratio of anesthesiologists per population, a workforce shortage is expected to worsen over the next ten years due to retirements, shortened call hours, and the Yatziv reform which bans graduates of certain overseas medical schools from obtaining Israeli Medical Licenses. The current workforce has compensated for the existing shortage of anesthesiologists by enlisting non-Israeli trainees from overseas. Yet, it is crucial to maintain and enlarge the local Israeli workforce to forestall a worsening shortage.

**Supplementary Information:**

The online version contains supplementary material available at 10.1186/s13584-024-00620-0.

## Introduction

Anesthesiology is a key specialty enabling the safe provision of anesthesia for surgery and analgesia for painful medical procedures. The contemporary anesthesiologist's role has expanded far beyond the confines of the operating rooms to labor and delivery rooms; gastroenterology, radiology, dental and electroconvulsive therapy suites; pediatric departments; intensive care units; acute and chronic pain units; and emergency trauma areas [1, 2]. This expanding role, when examined from the perspective of the number of employed physicians in the medical departments of Israeli hospitals, has resulted in anesthesiology departments being the largest or among the largest medical department [3]. Moreover, as medicine advances and the Israeli population continuously expands and grows older, the need for anesthesiologists is increasing, paralleling similar trends in other fields of medicine [4]. However, increasing the number of physician anesthesiologists depends on Israeli anesthesia residency programs attracting sufficient Israeli medical school graduates, Israeli citizens graduating from medical schools abroad, physician immigrants, and foreign graduates on temporary training visas. A study performed in 2005 showed that few Israeli medical school graduates selected anesthesiology as a career, with most anesthesiologists being immigrants from the former Soviet Union [3]. However, the number of immigrant physicians has since decreased substantially likely adding to a shortage of anesthesiologists, which limits the number of surgeries and other procedures in many hospitals [5]. The Ministry of Health thus designated anesthesiology as a specialty in crisis [5] which led to the 2011 salary agreement between the Israeli government and the Israel Medical Association to include one-time monetary grants for medical students choosing anesthesiology for residency training [6, 7]. This program was eventually terminated because overall funding was decreased and additional specialties required assistance. Despite this and other measures, the anesthesiology and healthcare leadership still recognize a shortage of anesthesiologists [5, 8, 9]. This shortage will be exacerbated if night duty is reduced from 24 to 16 h, removing some anesthesiologists from the daytime workforce [7]. It will be exacerbated further by the Yatziv reforms, which will reduce the number of overseas medical schools whose graduates will be able to obtain Israeli Medical Licenses [7]. This reform has already resulted in no Israeli students enrolling in these overseas medical schools since 2019.

This study examined the current state of the Israeli anesthesiology workforce, using and expanding upon the methodology from 2005. The aim was to characterize the composition of the present physician anesthesiology workforce, compare it to the 2005 workforce anddetermine whether the specialty has succeeded in attracting more Israeli medical school graduates.

## Methods

In March 2021, an anesthesiology workforce cross-sectional survey was conducted among the anesthesiology department chairs responsible for 36 Israeli acute care public and private hospitals. They were asked to provide information about each anesthesiologist employed in their department. The hospitals were identified using the Ministry of Health’s website and confirmed using information from the Israel Society of Anesthesiologists’ list of anesthesiology department chairs. Surveys were sent to the chairs via e-mail and involved completing data forms and a questionnaire. A review of the submitted replies by a non-physician research team member followed. Follow-up phone calls were made to all chairs who did not initially reply to request further information if questionnaire replies were incomplete or unclear. Data elicited about each anesthesiologist employed in their department included age, gender, employment status (full or part-time), academic medical school appointment, country of medical school education, country of anesthesiology residency, and current professional status (certified specialist or trainee). In addition, the department heads were asked to answer the question whether anesthesiology is a specialty suffering a workforce crisis. Note that in this paper, we refer to anesthesiology residents as *trainees* to differentiate them from *residents* – persons legally residing in a country where they are not citizens. We extended the data collected in the 2005 study to include citizenship status (citizen/permanent resident vs. non-citizen), subspecialty certifications and administrative appointments. In our analyses we focused on trainees under the age of 40 due to the phenomenon in Israel of considering many physicians who have failed the specialty examination on multiple occasions as "trainees" when they are really non-longer trainees but physicians who are not specialty certified.

We sourced Medline, the Israeli government (Ministry of Health, Central Bureau of Statistics) and the Internet to obtain information about the past, present, and projected future growth of Israel's population, its age distribution, and birth rate. The same sources provided immigration statistics, data on the physician workforce, and national healthcare activities. The number of anesthesiologists per 100,000 was calculated using population data from the Israeli Central Bureau of Statistics [10].

### Data analysis

Data were entered into Excel® (Microsoft Inc. Redmond WA) spreadsheets and statistical analysis was performed with SPSS® Ver. 26 (IBM Corp. Armonk NY).

Workforce quantitative characteristics were described as mean ± standard deviation, whereas categorical characteristics were presented as frequencies and percentages. To compare categorical variables to the 2005 study data, one-sample Chi-square tests were used. Testing the association between two categorical variables was performed using Chi-square tests. All tests were two-tailed, and a *p*-value ≤ 0.05 was considered significant. An in-depth analysis of the anesthesiologists with higher academic appointments (excluding clinical instructor and instructor and requiring approval by a promotions/appointment committee) as professor, associate professor, senior lecturer and lecturer was performed. This analysis included a logistic regression to examine the variables associated with a higher academic appointment.

The Institutional Review Board of Hadassah Medical Organization approved this study; completing questionnaires was an expression of implied consent.

## Results

**Fig. 1:**
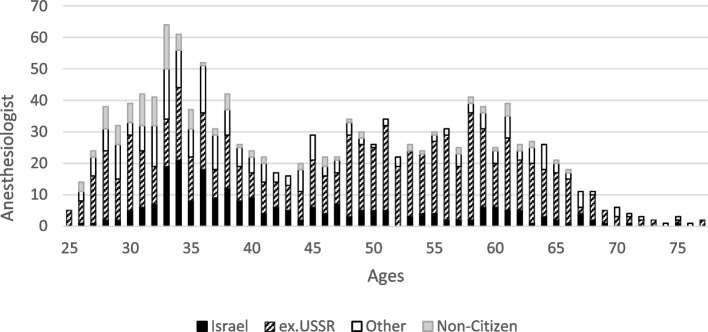
Age distribution of the 1313 hospital department anesthesiologists. The black bar represents the graduates of Israeli medical schools. The hatched bar represents graduates of former USSR medical schools who are Israeli citizens or permanent residents. The white bar represents graduates of other medical schools who are Israeli citizens or permanent residents. The gray bar represents non-Israeli citizens or permanent residents. The graph shows a U-shaped distribution with a higher prevalence of anesthesiologists between the ages of 55–65 years and 30–40 years, with a dip between the ages of 40–55 years

A 100% response rate was achieved among 34 Israeli anesthesiology department chairs who provided data on 36 (30 public, 6 private) hospitals (Supplementary Table). They reported on 1313 anesthesiologists compared to 711 anesthesiologists from 30 hospitals in the 2005 study. In the current study, based on a population of 9.291 million persons [5], there were 14.2 anesthesiologists per 100,000 people, while in 2005, there were 10.8 anesthesiologists per 100,000 population, an increase of 31.4%. Thirty-eight (2.9%) of the anesthesiologists were > 66 years old and 297 (22.6%) were between 57–67, i.e., they will reach the Israeli retirement age of 67 years within the next 10 years (Fig. 1). The characteristics of the anesthesiology workforce can be found in Table 1. Ninety-nine (7.5%) physicians worked part-time compared to 87 (12%) in 2005 (*p* < 0.001).

**Table 1:** Characteristics of the Israeli anesthesiology workforce

	**Trainees (Residents)**	**Attendings**	**Others**^**a**^	**Total**
N (%)	**536 (**40.8%)	**629** (47.9%)	**148** (11.2%)	**1313**
Age (years)	34.7 ± 6.8	52.7 ± 10.0	55.6 ± 9.7	45.5 ± 12.6
Range (years)	24–63	30–80	26–80	24–80
Females (n, %)	169, 31.5%	172, 27.3%	28, 18.9%	37, 28.7%
**Medical School Education**				
Africa	5 (0.9%)	10 (1.6%)	1 (0.7%)	16 (1.2%)
Eastern Europe	173 (32.3%)	102 (16.2%)	21 (14.2%)	296 (22.5%)
Israel	102 (19.0%)	115 (18.3%)	20 (13.3%)	237 (18.1%)
Middle East (non ISR)	43 (8.0%)	10 (1.6%)	2 (1.4%)	55 (4.2%)
North America	3 (0.6%)	13 (2.1%)	0 (0%)	16 (1.2%)
Russia	111 (20.7%)	278 (44.2%)	95 (64.2%)	484 (36.9%)
South America	25 (4.7%)	19 (3.0%)	3 (2.0%)	47 (3.6%)
Southern Europe	25 (4.7%)	27 (4.3%)	3 (2.0%)	55 (4.2%)
Western & Central Asia	25 (4.7%)	21 (3.3%)	2 (1.4%)	48 (3.7%)
Western Europe	20 (3.7%)	31 (4.9%)	1 (0.7%)	52 (4.0%)
Other	4 (0.7%)	3 (0.5%)	0 (0%)	7 (0.5%)
**Residency training**				
France		5 (0.8%)	1 (0.7%)	6 (0.5%)
Israel		601 (95.5%)	138 (93.2%)	1268 (96.6%)
Russia		7 (1.1%)	3 (2.0%)	13 (1.0%)
USA		11 (1.7%)	0 (0%)	11 (0.8%)
Other		5 (0.8%)	6 (4.1%)	15 (1,1%)

Data is presented as mean ± SD

^a^—Others – non- trainee non-certified specialist physicians who continue to work in a supervised capacity

The overall proportion of female anesthesiologists was 28.7% (Table 1), an increase of 12% from 25.0% in 2005 (*p* < 0.002). However, among trainees < 40 years old, there were only 137 (30.0%) females in 2021, a non-significant decrease from 31.1% in 2005 (Table 2). In the present study, 31.2% of the trainees < 40 years old who were Israeli citizens or Israeli residents were female, which is similar to the 2005 study (31.1%), where the cohort of non-Israel citizens or trainees was not a substantial factor. Notable among the non-Israeli citizens/non-resident anesthesiology trainees was the low proportion of females (22.9%).

**Table 2:** Demographic characteristics of anesthesiology trainees < 40

	**Israeli Citizens**^**a**^**, Trainees < 40**	**Non-Israeli Citizens**^**a**^**, Trainees < 40**	**All Trainees < 40**
n (%)	388 (85%)	70 (15%)	458
Age (year)	32.5 ± 3.6	31.7 ± 2.9	32.4 ± 3.6
Range (year)	24–40	26–40	24–40
Females (n, %)	121, 31.2%	16, 22.9%	137, 22.9%
**Medical School Education**			
Africa	0 (0%)	4 (5.7%)	4 (0.9%)
Eastern Europe	135 (34.8%)	12 (17.1%)	147 (32.1%)
Israel	91 (23.7%)	1 (1.4%)	93 (20.3%)
Middle East (non ISR)	29 (7.5%)	14 (20%)	43 (9.4%)
North America	1 (0.3%)	0 (0%)	1 (0.2%)
Russia	77 (19.8%)	3 (4.3%)	80 (17.5%)
South America	10 (2.6%)	12 (17.1%)	22 (4.8%)
Southern Europe	21 (5.4%)	2 (2.9%)	23 (5.0%)
Western & Central Asia	6 (1.5%)	16 (22.9%)	22 (4.8%)
Western Europe	15 (3.9%)	4 (5.7%)	19 (4.1%)
Other	2 (0.5%)	2 (2.9%)	4 (0.9%)
	*p* < *0.001, Chi-square test*		

^a^Citizens = Citizens and Permanent Residents

The overall proportion of Israeli medical school graduates in the anesthesiology workforce increased from 12.2% in 2005 to 18.1% in 2021 (*p* < 0.001). This observation becomes more marked when examining trainees < 40 years old. In 2005, 113 (73%) trainees < 40 years old studied medicine in the former Soviet Union, while only 13 (8.4%) studied in Israel. In 2021, 92 (23.7%) trainees were Israeli graduates, with only 77 (19.8%) coming from the Russian Federation. Instead, overseas medical school graduates in anesthesiology training programs studied elsewhere, in Eastern Europe (135/388, 34%) and non-Israel Middle Eastern countries (29/388, 7.5%).

The present study showed an influx of physicians coming to Israel for anesthesiology residency or fellowship training, with 111 (8.5%) of the anesthesiology workforce being non-citizens/non-residents working under provisional permits (Table 2).

In 2021 there were 3 full professors and 13 associate (including clinical) professors of anesthesiology, while in 2005, there were four full professors and 8 associate professors, demonstrating a decline from 3.2% (12 professors) to 2.5% (16 professors) of the anesthesiology attendings (NS). In 2021, there were 76 (12%) physicians with academic appointments, including professors, associate professors, senior lecturers, lecturers and instructors, a slight decrease in proportion from 66 (17.6%, *p* < 0.01) academic appointees in 2005.

A more in-depth analysis of the 55 anesthesiologists with higher academic appointments (excluding clinical instructors and instructors) as professor, associate professor, senior lecturer and lecturer was performed. They ranged in age from 33 – 75 years and were compared to the 567 specially certified anesthesiologists of the same ages (Table 3). Only 18 of the 28 public hospitals had anesthesiologists with higher academic appointments and none of the private hospitals. Sixty-two percent of the 55 anesthesiologists with higher academic appointments came from one of 4 hospitals. Logistic regression revealed the association of sub-specialty certification in critical care medicine (OR: 4.22; 95% CI: 2.17—8.21; *p* < 0.001) and an administrative position (OR: 1.60; 95% CI: 1.23—2.10; *p* < 0.001) with a higher academic appointment.

**Table 3:**
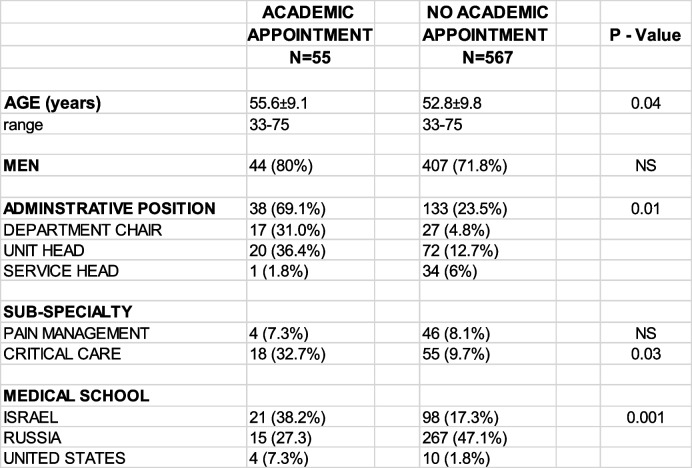
Higher Academic Appointments^a^

Values are mean ± SD

^a^Lecturer, Senior Lecturer, Associate Professor, Professor (including Clinical titles)

Among attending anesthesiologists, 69 (11%) are certified in critical care medicine and 59 (4.5%) in pain medicine. Among attending anesthesiologists, 168 (27%) have administrative appointments; 44 (7%) are ranked department chairs including ICU chairs or anesthesiology chairs, 90 (14.3%) are unit directors, and 34 (5.4%) are service directors.

All 34 department heads reported providing anesthesia outside the operating rooms. Twenty-nine of the 34 (85%) department heads believe anesthesiology is in a workforce crisis.

## Discussion

The 2021 Anesthesiology Workforce Survey provided much information on the current composition of the Israeli anesthesiology workforce. Comparing these new data to the 2005 survey highlighted that the workforce is still mainly composed of graduates of non-Israeli medical schools since relatively few Israeli medical school graduates enter the specialty. However, compared to 2005, the number of anesthesiologists per 100,000 population has grown by 31.4%, from 10.8 to 14.2, which contrasts with the overall decrease in physicians per 1,000 population from 3.6 in 2005 to 3.3 in 2020 [11, 12]. This increase in anesthesiologists might be an encouraging indication that the anesthesiology workforce has increased in both absolute and relative terms, thus diminishing the national shortage of anesthesiologists. However, 14.2 anesthesiologists per 100,000 population remains significantly lower than most developed countries: France—15.06, UK—17.8, US—20.8, Russia—20.9, Norway—25.5, Germany—30.9 (2015 data from the Global Anesthesiology Workforce Survey) [13]. It is important to note that in the US and France, the physician anesthesiology workforce is further augmented by anesthesia extenders, such as nurse anesthetists and anesthesia assistants [14]. Notably, the anesthesiologist workforce shortage persists, as shown by the 2021 survey where 85% of department heads believe anesthesiology suffers from a workforce crisis and data from a 2019 survey where 21 of 34 department heads (62%) reported that operating rooms are routinely closed, or procedures canceled in their hospitals due to workforce shortages [6]. This situation is similar to that of specialties, such as emergency medicine, which have an increasing physician-per-population ratio but are experiencing workforce shortages [15].

Despite the absolute and per capita increases in anesthesiologists, the persistent workforce shortage can be explained by the ever-increasing demand for anesthesia services. The Israeli population grew from 6.91 million in 2005 to 9.29 million in 2021, while the population ≥ 65 years increased from 675,000 in 2005 (9.7%) to 1.145 million in 2021 (12.3%) [10]. These increases were major contributors to the increase in surgeries from 625,128 (estimated) in 2005 to 929,498 (estimated) in 2021 (C. Weissman, Personal Communication). Another contributing factor is the 22% increase in births (145,000 in 2005 vs. 177,000 in 2021), which results in both higher obstetric anesthesia (epidural catheter placements and cesarean sections) and pediatric anesthesia demands. A further reason for the greater anesthesia workload is the demand for non-operating room anesthesiology (NORA) services, such as gastroenterology, bronchoscopy, and cardiac catheterization procedures. All the anesthesiology department heads reported providing NORA services. In 2014, in the United States' 35% of anesthetics occurred in non-operating room settings and are expected to reach more than 50% of all anesthesia services by 2025 [1, 16]. Therefore, the demand for anesthesia services in Israel would be expected to increase at a rate greater than that estimated from only the increase in population, further contributing to the ongoing anesthesiologist shortage.

The distribution of anesthesiologists' ages has a U-shaped pattern. The older age peak was expected since it reflects the rise in anesthesiologists between the ages of 40–45 observed in the 2005 study caused by the wave of immigrant physicians from the former Soviet Union in the 1990s, a group now approaching the mandatory retirement age of 67 years. In most government, Clalit Health Services and public hospitals 67 years is the retirement age. However, in some of these hospitals and in the private hospitals a minority of anesthesiologists continue to work past the age of 67 years as reflected by the data presented in this article. This retirement age explains the sharp decline in practicing anesthesiologists > 67 years. Unless the retirement age is increased, 22.6% of the workforce will retire over the next ten years. The younger age peak appears to be mainly due to increases in both Israeli medical school graduates and non-citizens coming to Israel for training. The increase in Israeli medical school graduates is likely the result of the one-time monetary grants introduced in the 2011 wage agreement, which were terminated a few years later. More research is thus warranted to determine if reinstating one-time grants would continue to draw local graduates to the specialty. Such research is timely since recent increases in the number of Israeli medical students will increase the pool of Israeli graduates from which to recruit anesthesiology trainees [5, 8, 12].

This study demonstrates a slight decline in the number of anesthesiologists with academic positions. Furthermore, there were many public hospitals without anesthesiologists at or above the rank of lecturer. This situation is not tenable in an environment where the number of Israeli medical students is steadily increasing causing a need for more clinical sites for medical student instruction, as well as the need for existing sites to take on greater teaching loads. Therefore, programs to encourage more anesthesiologists to pursue academic careers are urgently needed, such as more funded academic and administrative positions, additional research opportunities for attending anesthesiologists and funded post-residency research training fellowships.

Non-Israeli citizens and non-Israeli residents training in anesthesiology represents a substantial percentage of the current workforce (8%) and a more significant portion of the trainees < 40 years old (15.2%). The physicians who are not Israeli residents can be divided into two groups: Residents from the Palestinian Authority who are in anesthesiology residencies in Israeli hospitals. They usually return to hospitals in the Palestinian Authority upon completing their residencies or continue to fellowship training abroad. The other group come mainly from South and Central America countries which lack sufficient residency positions for all their medical school graduates or from the Central Asian Republics (former Soviet Union countries such as Georgia) which do not provide enough positions or the quality of training these physicians want. These residents are very motivated and provide clinical services equal to those provided by Israeli medical school graduates. This has been a way to maintain and slightly increase the anesthesiology workforce in the face of an inadequate local workforce. Importantly, these non-citizen physicians are not expected to remain in Israel over the long term and thus do not provide a long-term solution to reducing the workforce shortage unless the recruitment of such short-term trainees continues and even increases over the long term. Whether more non-Israeli residents can be attracted to Israeli anesthesiology training programs is an issue that should be explored on a national level, especially since some countries lack sufficient training programs for all their medical students and are seeking trainee positions for their students in other countries. However, this option might not be completely viable since the Yatziv Reforms will likely reduce the number of non-Israeli trainees, since some are graduates of medical schools that are not approved by the Israeli Ministry of Health.

The current study showed that 81.9% of all anesthesiologists and 79.7% of trainees under 40 studied medicine abroad, mostly in Russia and Eastern Europe. This proportion exceeds the 64% of new licensees (in 2020) who graduated from non-Israeli medical schools [12]. This situation is especially problematic since the Yatziv Reform will limit the number of foreign graduates receiving Israeli licenses [7].

In 2020, female physicians comprised 41.9% of Israeli physicians [12], substantially higher than the proportion of female anesthesiologists (28.7%) reported in this study. This is likely due to the high proportion of non-Israeli graduates who are predominately men. Although the proportion of female anesthesiologists has increased since 2005, the workforce continues to suffer from the underrepresentation of women. This is likely due to the perception that anesthesiology as a specialty does not enable an acceptable work-life balance [17]. As the proportion of Israeli medical school graduates’ increases, research is needed to determine what can be done to improve the field's attractiveness to women. The diversity of the anesthesiology workforce is important [18]. There is likely little net impact of gender on the total effective workforce; more maternity leave and possibly lower availability for long overtime hours among women [4, 19] balanced against more military reserve duty among men. However, with women becoming a greater percentage of the physician workforce it will be necessary to examine the effects on the anesthesiology workforce and its productivity.

### Strength and limitations

This study’s strength is its use of quantitative data collected from all the private and public hospitals, thus providing a comprehensive picture of the situation facing Israeli anesthesiology. Furthermore, we revisited a study conducted 15 years ago using the same data format, allowing us to compare changes over time. The limitation of this study is that it only provides information on one specialty and uses self-reporting by anesthesiology department chairs as the predominant source of information. However, much of their data has been corroborated by publicly available data from the Central Bureau of Statistics, the Ministry of Health, and the State Comptrollers Report. Another limitation is that only hospital departments were queried and thus did not include the anesthesiologists who work in non-hospital settings such as dental offices and gastrointestinal endoscopy centers thus under-counting the number of anesthesiologists. Since the data was collected without the names and/or identity numbers it was not possible to definitively cross-check names between different department heads and might have over-counted the number of anesthesiologists since a few might have worked part-time at more one institution.

We used the data that we collected from the anesthesiology department heads to calculate the number of anesthesiologists per 100,000 population because it is a number that we can rely on. When we examined the data from the 2020 Ministry of Health report (published in July 2021) on medical and nursing professionals we found that it reported 749 certified anesthesiologists below the age of 67 years in a number of graphs and tables and 1003 in another location (13). It is estimated that 10–15% of anesthesiologists certified in Israel are practicing outside of Israel so the number of certified specialist anesthesiologists practicing in Israel using the Ministry's data should be around 637 to 674 (6). This number is similar to the 629 certified specialist anesthesiologists reported by the anesthesiology department heads. Therefore, it would appear that around 45 certified anesthesiologists are engaged exclusively (outside of anesthesiology departments) in administrative, pain management, dental, critical care or private practice is very plausible. These additional anesthesiologists would only minimally change the number of anesthesiologists per 100,000 population.

## Conclusions and policy implications

Despite increases in the absolute number of anesthesiologists over the past 16 years, a workforce shortage persists. With continued growth in surgeries, births, and non-operating room anesthetics, this shortage will worsen over the next ten years, especially, with the retirement of 22% of the workforce. Further accentuating the shortage is the reduction of on-call shifts from 26 to 18 h and the Yatziv reform restricting foreign medical graduates from non-accredited schools from obtaining an Israeli license. The enlisting of foreign non-citizen trainees, which has compensated for some of the existing shortage, is insufficient, probably unsustainable, and is particularly vulnerable to the Yatziv reform. These factors threaten the future ability of the Israeli healthcare system to provide adequate anesthetic services. Therefore, the results of the current study support the findings of the recent OECD report which warned of the impending decrease in the number of Israeli physicians per capita and the need to train more physicians in Israeli medical schools, as well as subsidize the training of others in foreign schools [20]. The report also recommended that the current haphazard hospital/department-based method of allocating the number of residency positions and the number of positions per specialty be replaced by a more centralized needs and data-driven allocation of residency positions overall, by specialty and by hospital. Therefore, concerted governmental and professional efforts are promptly needed to provide the resources and conditions for not only sustaining but enlarging the indigenous Israeli anesthesiology workforce. These efforts will require ascertaining why so few Israeli medical school graduates choose anesthesiology as a career medical specialty and then using these data improve the attractiveness of the specialty. For example. these efforts could build on a small methodology-focused study which queried medical student’s and intern’s opinions as to why anesthesiology was not an attractive specialty and then explored what possible steps could be taken to improved its attractiveness [21]. Such evidenced-based and data-driven initiatives that address how to attract medical students to specialties suffering workforce shortfalls are thus an imperative since not only does one need to train sufficient numbers of physicians but failure to train sufficient specialists in vital specialties, such as anesthesiology, will affect the future ability of the Israeli healthcare system to provide its vaunted excellent care to the Israeli population.

## Supplementary Information

Supplementary Material 1.

## Data Availability

All data generated or analyzed during this study are included in this published article.
