# Supplementary material for: The Israeli anesthesiology workforce crisis: a reassessment survey
Source: Isr J Health Policy Res. 2024 Sep 17;13:48. doi: 10.1186/s13584-024-00620-0 (PMC11406861; doi:10.1186/s13584-024-00620-0)
Supplement: Supplementary file 1 — Supplementary Material 1. [file 13584_2024_620_MOESM1_ESM.docx]

**Supplementary Table – List of all Israeli hospitals and whether included or not included.**

Al-Makassed Hospital, Jerusalem (P) - not included in ISA database

Al-Quds Maternity Hospital, Jerusalem - not included (PR) – maternity only

Assuta Hospital, Beersheba (PR)

Assuta Ramat HaHayal, Tel Aviv (PR)

Assuta Hospital, Rishon Lezion (PR)
Assuta Hospital, Haifa (PR)

Augusta Victoria Hospital, Jerusalem - not included in ISA database

Baruch Padeh Medical Center, Poriya (G)
Barzilai Medical Center, Ashkelon (G)

Bnai Zion Medical Center, Haifa (G)

Carmel Medical Center, Haifa (C)
Edith Wolfson Medical Center, Holon, (G)
Elisha Medical Center, Haifa (PR)

Emek Medical Center, Afula (C)

EMMS Nazareth Hospital, Nazareth (P)

French Hospital, St Vincent de Paul, Nazareth (P)

Galilee Medical Center, Nahariya (G)
Hadassah Ein Kerem & Mt, Scopus Medical Centers, Jerusalem (P)

Herzlia Medical Center, Herzlia (PR)

Hillel Yaffe, Hadera, Israel (G)
Horev Medical Center, Haifa (PR) - not included - low volume of surgeries

Italian (Holy Family) Hospital, Nazareth (P)

Kaplan Medical Center, Rehovot (C)
Laniado Hospital, Netanya (P)

Mayanei Hayeshua Medical Center, Bnei Brak (P)

Meir Medical Center, Kfar Saba (C)

Rabin Medical Center, Campus HaSharon Petach Tikva (C)
Rabin Medical Center, Campus Beilinson, Petach Tikva (C)

Rambam Medical Center, Haifa (G)
Raphael Hospital, Tel Aviv (PR) - not included - low volume of surgeries

Rebecca Sieff (Ziv) Hospital, Safed (G)

Red Crescent Maternity Hospital (P) - not included – maternity only

Saint John Eye Hospital, Jerusalem (P) - not included – only ophthalmology

Saint Joseph Hospital, Jerusalem (P) - not included in ISA database

Schneider Children's Medical Center, Petach Tikva (C)
Samson Assuta Ashdod Medical Center; Ashdod (P)
Shaare Zedek Medical Center plus Bikkur Cholim, Jerusalem (P)
Shamir Medical Center (Asaf HaRofe), Rishon LeTzion (G)

[Sheba Medical Center, Tel-HaShomer](about:blank) (G)
Soroka Medical Center, Beersheba (C)
Tel Aviv (Ichilov) Medical Center; Tel Aviv (G)
Yoseftal Medical Center, Eilat (C)

* C = Clalit Health Fund-owned; G = Government-owned; P = Public Hospital – owned by non-profit organization; PR – Private Hospital; ISA = Israel Society of Anesthesiology
